# Active Biodegradable Starch/PBAT-poly(butylene adipate-co-terephthalate) Film with *Eucalyptus citriodora* Essential Oil Incorporation

**DOI:** 10.3390/foods13132104

**Published:** 2024-07-02

**Authors:** Juliano Zanela, Marianne Ayumi Shirai, Juliana Bonametti Olivato, Maira Casagrande, Cristian Medrado Canonico, Américo Wagner Júnior, Fabio Yamashita

**Affiliations:** 1 Department of Food Science and Technology, State University of Londrina, Rod. Celso Garcia Cid (PR 445), Km 380, P.O. Box 6001, Londrina 86051-990, PR, Brazil; fabioy@uel.br; 2 Agronomy School, Federal University of Technology—Paraná, Estrada para Boa Esperança, Km 04, Dois Vizinhos 85660-000, PR, Brazil; mairacasagrande1@gmail.com (M.C.); cristianm.canonico@gmail.com (C.M.C.); americowagner@utfpr.edu.br (A.W.J.); 3 Department of Food Technology, Federal University of Technology—Paraná, Londrina 86036-370, PR, Brazil; marianneshirai@utfpr.edu.br; 4 Pharmaceutical Sciences Department, State University of Ponta Grossa, Av. General Carlos Cavalcante, 4748, Ponta Grossa 84030-900, PR, Brazil; jbolivato@uepg.br

**Keywords:** extrusion, antioxidants release, food simulants, packaging

## Abstract

Plastic pollution and the reduction in synthetic food additives are demands that emerge from consumers, leading to the development of biodegradable plastic materials. The use of essential oils—EOs—has been researched because it is a natural product with antioxidant properties. Due to its nature, EO is composed of volatile compounds that can be lost during extrusion. The aim of this work was to produce active biodegradable starch/PBAT films with the incorporation of neat Eucalyptus citriodora EO (0.5, 1.0, and 1.5%) or EO microencapsulated by spray drying (2.5, 5.0, and 7.5%), aiming at the protection of the EO. The produced films showed adequate mechanical properties (tensile strength ranged from 5.72 to 7.54 MPa and the elongation at break ranged from 319 to 563%). Testing in food simulants showed that the films retained antioxidant activity, being more suitable for use in fatty or non-acid foods, with the microencapsulation process offering protection to the EO during the process.

## 1. Introduction

Consumers have increasingly searched for more natural or healthy foods, and plant extracts or natural additives are generally accepted as substitutes for synthetic agents due to concerns about the latter’s deleterious effects on human health [1,2]. Inside the group of natural compounds with biological activity are the essential oils (EOs). EOs are aromatic compounds formed by diverse organic compounds such as terpenoids and phenolic compounds. These chemical compounds have antimicrobial and antioxidant properties and can control foods’ microbial growth and lipid oxidation processes. Due to these properties, they present great potential for application in active films [3,4,5].

*Eucalyptus citriodora*, also known as *Corymbia citriodora*, among other names, is one of around 900 species and subspecies that pertain to the Myrtaceae family of Eucalyptus. In addition to the production of wood and cellulose, the essential oils obtained from their leaves are used in medicine, perfumery, and the food industry. In this case, they are valued due to their antibacterial, antioxidant, and flavoring properties [6,7,8].

The volatility of EOs can be a critical issue to be considered in their application. A proportion of 90–95% of the EO is composed of volatile substances that can lead to losses in concentration. Compound oxidation is another problem affecting the EO, mainly in monoterpenes. A way to overcome these drawbacks is the encapsulation of the EO, which can protect it from external factors, such as the temperature during food processing, for example [5]. Encapsulation by spray drying is commonly used to preserve the active compounds. It can be described as a mechanical or physicochemical process of entrapping the active molecules in a bulk composed of a wall material [9] that protects the active compounds during production operations.

With this trend in vogue, active packaging can emerge with potential use because it removes the active agents from the food, transferring them to the packaging material. The use of active packaging can increase the food’s shelf-life, modifying the environment or metabolic function of packaged food and maintaining its freshness for a longer time, with several materials presenting compatibility and biodegradability for use [10,11].

Another emerging preoccupation is the pollution caused by the inadequate disposal of plastic waste, which harms the environment and living organisms. Plastic packaging is a widely used material in industry to protect and prolong the shelf-life of foods. However, these plastics are commonly petroleum-based polymers that are non-biodegradable and non-renewable. In the food sector, there is still the problem of the overuse of single-use packages [12,13]. The above-related problems urge the development of biodegradable materials and, if possible, fully renewable sources, such as starch.

Starch has a broad potential for replacing petroleum-based, non-biodegradable polymers as packaging materials. Due to being low-cost, fully renewable, biodegradable, and widely available, they produce odorless and non-toxic films that act as food contact materials. However, starch-based materials present some drawbacks related to their hydrophilic nature, which makes them highly sensitive to moisture, resulting in poor mechanical and barrier properties [14,15,16].

A way to overcome the drawbacks of starch is to blend it with other polymers, resulting in materials with adequate properties for the desired application. One of these polymers is the PBAT-poly(butylene adipate-co-terephthalate). PBAT is a biodegradable polymer with similar proprieties to LDPE (low-density polyethylene) and is commercially available, but at a higher price compared to conventional non-biodegradable polymers [17]. The production of blends by thermoplastic extrusion between starch and PBAT has been made with relative success [14,16,18]. Starch reduced the cost of the produced material, while PBAT increased the mechanical and barrier properties, resulting in materials with adequate properties for use.

This work aimed to develop an active biodegradable film based on cassava starch/PBAT, with the *Eucalyptus citriodora* essential oil as the active agent, through blown-film extrusion. The neat or microencapsulated EO was added by spray drying to evaluate the loss and the release of the EO in different incorporation methods when in contact with food simulants.

## 2. Materials and Methods

### 2.1. Materials

The *Eucalyptus citriodora* essential oil was purchased from a producer industry (Quinarí, Ponta Grossa, Brazil). Arabic gum (Instantgum BB, Nexira, São Paulo, Brazil) and maltodextrin (Mor-rex 1920, with a 20-dextrose equivalent value, Cargill, Porto Ferreira, Brazil) were the wall material components for the microencapsulation process. The extruded blown film was produced using cassava starch (Yoki, São Paulo, Brazil) and PBAT-poly (butylene adipate co-terephthalate) (Ecoflex^®^ F BX 7011, BASF, Lemförde, Germany). Glycerol (Dinâmica, Indaiatuba, Brazil) was used as the plasticizer. Other chemical reagents used in the analysis were reagent grade.

### 2.2. Methods

#### 2.2.1. Eucalyptus Citriodora Microencapsulation by Spray Drying

The spray-drying conditions were set after pre-tests. The wall material composition is based on a 75:25 blend of Arabic gum: maltodextrin. The EO was added in a proportion corresponding to 20% of the total solids, and the final solids concentration of the emulsion was 30% (*w*/*v*%). After complete solubilization of the wall materials, the EO was added, and the emulsion was formed in a homogenizer (TE-105, Tecnal, Piracicaba, Brazil), operating at 13,000 RPM for five minutes.

The microcapsules were produced in a lab-scale spay dryer (SD-05, Labplant, Filey, UK). The inlet temperature was set at 180 °C, and the outlet temperature at 115 °C. The emulsion feed flow of 450 mL·min^−1^ was used. The powder was collected and maintained in a sealed flask until use.

The encapsulation efficiency was determined using Clevenger’s extraction apparatus [19].

#### 2.2.2. Production of Extruded Active Biodegradable Blown Film

The control film composition was 46, 40, and 14% (*w*/*w*) of starch, PBAT, and glycerol, respectively. The neat EO was added in proportions of 0.5, 1.0, and 1.5% (samples named 0.5% EO, 1.0% EO, and 1.5% EO, respectively). The microencapsulated EO was added in the proportions of 2.5, 5.0, and 7.5% (samples named 2.5% MC, 5.0% MC, and 7.5% MC, respectively). The mass of neat or microencapsulated EO was discounted from the starch mass.

The mass of the constituents of each film was manually homogenized and extruded for pellet production in a single-screw extruder (EL-25, BGM, São Paulo, Brazil) with a 25 mm screw (L/D ratio = 30), a screw speed of 35 rpm, and a temperature profile of 90/120/120/120 °C from the feeder to the die. The pellets were processed in the same equipment but with a 50 mm film-blown die and temperatures set at 90/120/120/130 °C from the feeder to the die, with a screw speed of 35 RPM.

#### 2.2.3. Eucalyptus Citriodora CG-MS Analysis

The composition of the EO was determined in a gas chromatograph–mass spectrometer QP-2010 SE (Shimadzu, Kyoto, Japan), equipped with a fused silica capillary column RTX-5 (Restek, Bellefonte, USA) (length of 30 m, i.d.: 0.25 mm, film thickness: 0.25 μm); helium was used as the carrier gas at a flow rate of 1.2 mL·min^−1^. The EO was diluted in hexane, and 1 μL (split mode, 1:10) was injected at 250 °C.

The initial column oven temperature was 60 °C (held for 2 min), followed by a heating rate of 3 °C·min^−1^ until it reached 240 °C, with the heating rate increasing to 10 °C·min^−1^ until 280 °C, holding for 10 min at this temperature. The fragments were compared with NIST mass spectra libraries. A duplicate was performed.

#### 2.2.4. Release of Total Phenolic Compounds—TPCs and ABTS Cation Scavenging Assays in Food Simulants

The TPC content was determined through Folin-Ciocalteau’s spectrophotometric method [20]. The antioxidant activity by ABTS cation scavenging was performed, too [21,22]. These analyses were performed in quadruplicate.

The food simulants were chosen according to the European Union regulation (N° 10/2011) related to the plastic materials in contact with foods. We chose food simulants A (ethanol 10% *v*/*v*) and B (acetic acid 3% *v*/*v*), which are relevant to hydrophilic foods, with food simulant B representing foods with a pH lower than 4.5. The food simulant D1 (ethanol 50% *v*/*v*) simulates lipophilic foods and oil-in-water emulsions. Distilled water was used, too.

The extraction and quantification of the TPCs and the antioxidant activity of the films in the food simulants were determined according to Talón et al. (2017), with some modifications [23]. Film samples were cut into small bits, weighted (1.5 g), and added in separate conical centrifuge tubes, each containing 25 mL of a food simulant. The tubes were sealed and kept in a water bath at 25 °C for 20 h. The extract was filtered and analyzed as described above.

#### 2.2.5. Mechanical Properties

The tensile strength, Young’s modulus, and elongation at break were determined according to the modified ASTM D638-03 method. Ten specimens of each sample were cut in a longitudinal stretching direction and previously conditioned (relative humidity of 53 ± 2% and temperature 23 ± 2 °C) for one week and analyzed in a universal testing machine (DL2000, EMIC, São José dos Pinhais, Brazil), with a crosshead speed of 0.8 mm·s^−1^ and an initial distance between the grips of 40 mm.

#### 2.2.6. Optical Properties

The apparent opacity (Op) and color difference (ΔE*) were determined using a colorimeter (BYK Gardner, Geretsried, Germany) with a D65 illuminant (daylight) and a visual angle of 10° [24], with five replicates for each sample. For the ΔE* analysis, the values of a white pattern calibration ceramic (L* = 94.31, a* = −1.52, and b*= −0.63) were used, with the differences observed in the films related to this white pattern.

#### 2.2.7. Swelling Index (SI) and Weight Loss in Water (WLW)

Both analyses were determined in triplicate. The SI was determined as the percentual mass of water that the film was able to retain after staying immersed for 24 h in water [25]. The WLW was determined gravimetrically as the percentage of mass lost by the film after 48 h of immersion in water [26].

#### 2.2.8. Water Vapor Permeability—WVP

The WVP was determined in the relative humidity gradient of 33–64%, according to the gravimetric ASTM E96-009 method.

#### 2.2.9. Fourier Transform Infrared Spectroscopy—FT-IR

The films were previously dried in a desiccator containing a calcium chloride salt. The spectra were recorded in a 4000 to 750 cm^−1^ spectral range in an FT-IR (IR Prestige 21, Shimadzu, Kyoto, Japan).

#### 2.2.10. X-ray Diffraction—XRD

The diffractograms were collected in a diffractometer (X’Pert PRO MPD, Panalytical, Malvern, The Netherlands), at room temperature and between 2θ = 5–50°.

The crystallinity index was determined as the ratio between the strongest peaks (crystalline region) by the amorphous area (area under the curve).

#### 2.2.11. Scanning Electron Microscopy—SEM

Dried films (over calcium chloride) were fractured in liquid nitrogen and gold-coated (BAL-TEC SCD 050, Leica, Wetzlar, Germany), and the fracture surface micrographs were recorded on a scanning electron microscope (Quanta 200, FEI, Hillsboro, USA).

### 2.3. Statistical Analysis

Data were analyzed by performing an analysis of variance (ANOVA) and Tukey’s test (*p* < 0.05) in STATISTICA 14.0 software (TIBCO, Palo Alto, USA).

## 3. Results and Discussion

Both microencapsulation processes were successfully used in the film production.

The microcapsules present a pale-yellow color, with a characteristic Eucalyptus odor, and the microencapsulation efficiency was 44.02%. The efficiency is lower than that obtained for our research group for clove essential oil in similar conditions (60.95%). This yielding can be due to the higher volatility of citronellal [27], which causes a higher loss of compounds during the process, reducing the percentage of EO in the microcapsules.

The produced films are concise and cohesive, with good handling and processability, presenting a whitish color and a smooth texture, and all the films present an average thickness of 172 ± 27 µm.

### 3.1. Eucalyptus Citriodora CG-MS Analysis

The EO was mainly composed of citronellal (72.81%), followed by citronellol (6.99%), isopregol (5.72%), β-citronellal (3.58%), and citronellol acetate (1.58%); these five components represent 90.58% of total EO composition.

These values are according to the literature, with contents of citronellal and citronellol found to be 68.91 and 7.6% [28], 62.4 and 10.6% [29], respectively. These variations in the EO composition are normal and expected in plant metabolites due to genetic and climatic conditions. The content of citronellal ranged from 87.4 to 69.7%, and that of citronellol ranged from 9.9 to 5.1% during one year of monitoring of *Eucalyptus citriodora* EO grown in the sub-tropical conditions of North India [30].

### 3.2. Release of Total Phenolic Compounds (TPCs) and ABTS Cation Scavenging Assays in Food Simulants

The neat EO presented an ABTS value of 12.49 µmol TEAC·g^−1^, and an EC_50_ value of 3.68 mg·mL^−1^. Values of EC_50_ lower than 50 µg·mL^−1^ indicate that the sample can be considered to have high antioxidant activity [31], so the obtained value for the *E. citriodora* EO (3.68 mg·mL^−1^) shows the potential of this EO for application as an antioxidant agent.

The obtained values are higher than those in the literature. Amri et al. (2023) obtained an EC_50_ value of 0.05 mg·mL^−1^ and Miguel et al. (2018) obtained an ABTS value of 5.08 µmol TEAC·g^−1^ [7,32]. Both authors cited previously observed that *E. citriodora* was the EO with higher antioxidant activity compared to other Eucalyptus species in their respective works, indicating the potential use of this species in particular. These variations can be due to natural differences in the EO composition. Citronellal, the main component of EO, does not present conjugated double bonds that result in a weaker free radical scavenging potential; however, it is possible to observe synergistic effects between the components, such as citronellal with α-phellandrene or p-cymene [33], which may explain the observed differences in the results. This study reinforces the need for a case-to-case evaluation because this is a complex system, with the possibility of many synergistic or antagonist combinations due to the natural variation in the EO composition.

Table 1 presents the results for the ABTS and CFT values in the films in contact with different food simulants. For the different films in each food simulant, in general, the films with 1.5% EO, 7.5%, and 5.0% MC presented higher values for both ABTS and CFT. The higher values observed for 7.5% MC and 5.0% MC films indicate that the microencapsulation process was able to protect the EO. This is evident because the real EO concentration is lower when compared to the films with pure EO added in the films. Observing the same film in different food simulants, ethanol 50% shows the higher values, with the distilled water and acetic acid 3% presenting the lower results.

The higher results shown in 50% ethanol can be due to this solvent presenting a more hydrophobic character, with a similar polarity to the EO components. The same was observed in chitosan films with bergamot essential oil through the evaluation of limonene release (the main component of this EO) in simulants that ranged from pure water to 95% ethanol solution, with the increase in the ethanol percentage in the solution. Consequently, the more the polarity decreased, the more limonene was released from the film. However, more than that, the authors observed that the hydration rate of the polymer is a driving factor, too [34].

The antioxidant and phenolic compounds quantified are correlated to the content of the EO in the films, similar to those observed in starch foams added with spent coffee grounds and oregano EO. According to the authors, the release process is dependent on the diffusion process in the polymeric matrix, which consists of three steps: firstly, the diffusion of the solvent in the polymeric matrix, which causes solvation and plasticization of the polymer network, promoting its relaxation, and finally, the diffusion of the active component. This process is affected by the pH and polarity of the solvent [35].

This demonstrates that the process is complex and not easily explained without an experimental study. As observed mainly for the CFT, the solvent’s acidification reduced the release compared to pure water, probably due to the lower relaxation of the polymeric network caused in this condition.

The real EO content in the microencapsulated EO films was 0.22, 0.44, and 0.66% for 2.5% MC, 5.0%, and 7.5% MC, respectively, being lower than in the films with neat EO (0.5, 1.0, and 1.5%). However, the results observed in the food simulants are similar, despite the lower content of EO in the microencapsulated films, demonstrating that the spray-drying process was effective in protecting the EO during the high temperatures of the extrusion process and was able to release it posteriorly.

### 3.3. Mechanical Properties

The results for the mechanical properties of the produced films are summarized in Table 2. The presence of neat or microencapsulated EO causes alterations in the film properties.

The film with 0.5% EO shows better values for tensile strength and elongation at break, and the films with microcapsules lead to the increase in Young’s modulus. The opposite occurs for tensile strength and elongation at break in the presence of the microcapsules, leading to a tendency of reduction in the mechanical properties.

It is commonly observed that the incorporation of pure essential oil acts as a plasticizer, causing an increase in elongation at break. In this work, the 0.5% EO shows this behavior. In higher concentrations, depletion of mechanical properties occurs. Instead, the microcapsules usually act as fillers, reducing the mechanical properties probably due to the presence of the stress concentration points. This behavior was observed in cassava starch/PBAT films with neat and microencapsulated oregano EO (Medeiros et al., 2019) [36]. Similar behavior was observed in corn starch with microencapsulated thyme EO, showing decreased elongation at break with increased microcapsule content [37]. Talón et al., (2019), observed a reduction in the mechanical properties of compression-molded films of corn starch with free and microencapsulated eugenol oil [38]. In the microencapsulated materials, the authors claim that the reduction is due to the presence of discontinuities in the polymeric matrix.

However, despite the reduced mechanical properties, the films still have good characteristics that justify their potential application as food packaging.

### 3.4. Optical Properties

The apparent opacity ranges from 63.3 to 66.6%, and the ΔE* ranges from 10.5 to 12.7. They indicate a lower variation despite the presence of EO. All the films are whitish and opaque, resulting in high opacity values. The extrusion process leads to the higher opacity values, due to the biaxial elongation of the material during the blown-film formation, which favors the formation of crystalline zones, increasing the film’s opacity [19].

The presence of microcapsules leads to a light yellowish of the films, increasing the ΔE* values in the films containing microcapsules, as observed in Table 3.

The obtained values agree with the literature. An opacity of 65.88% and an ΔE* value of 10.14 were found in blown film extruded from cassava starch/PBAT [39]. The presence of free or microencapsulated oregano EO in wheat flour/PBAT-extruded films causes an increase in the opacity values, too, ranging from 58.27 to 64.19% [40].

### 3.5. Swelling Index (SI) and Weight Loss in Water (WLW)

The SI values range from 19.45 to 32.38% (Table 3). The increase in the EO content in both incorporation forms leads to reduced values, with the higher reduction occurring in the neat EO films.

The reduction in SI values can be attributed to the higher hydrophobicity of the films with increased EO content [41]. This explanation is valid in the case of neat essential oil, which presents droplets of dispersed EO. In the case of the film containing microcapsules, the lower values can be attributed to the higher WLW values for this film, which consequently reduce the mass and the capacity of the polymer to swell.

The higher values of WLW were observed in the films containing microcapsules, with the values ranging from 14.57 to 18.14% for all films. These values are higher than those observed by Balan et al. (2021) in wheat flour/PBAT-extruded films with microencapsulated oregano EO, where the values ranged from 4.25 to 14.30% for the control and the film with 10% of microcapsules added, respectively [40]. According to the authors, this can be related to the hydrophilicity and solubility of the microcapsule wall material composition (Arabic gum and maltodextrin—the same used in the present work). The solubilization of the microcapsules and the lixiviation for the water promote an increase in the WLW values in these films.

### 3.6. Water Vapor Permeability—WVP

The WVP analysis does not show statistical differences between the films, with a mean of 7.1 × 10^−10^ g·m^−1^·s^−1^·kPa^−1^, as shown in Table 3. These values agree with other works based on starch/PBAT films: a WVP value of 6.1 × 10^−10^ g·m^−1^·s^−1^·kPa^−1^ [39] and of 2.05 × 10^−7^ g·m^−1^·h^−1^·Pa^−1^ [19]. These data demonstrate that the EO could not cause a significant alteration in the water molecule’s flux through the polymer net. The presence of some holes and cracks observed in the SEM analysis (section below) can explain this. These spaces in the polymeric matrix can make the transport of water vapor easier [42], resulting in no difference in the presence or absence of EO.

### 3.7. Fourier Transform Infrared Spectroscopy—FT-IR

**Figure 1:**
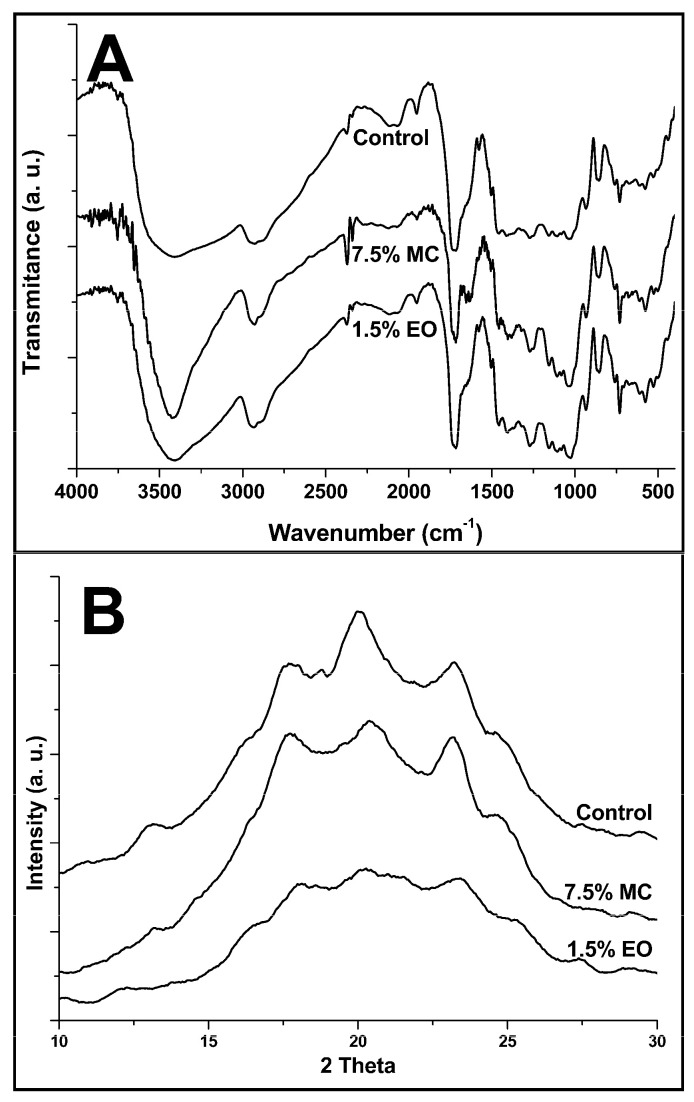
FT-IR spectra (**A**) and diffractograms (**B**) of the active biodegradable films containing neat or microencapsulated *E. citriodora* essential oil.

The FT-IR spectra are shown in Figure 1A. It is possible to observe that the incorporation of EO does not promote significant differences among the spectra. The broad band observed near 3300 cm^−1^ is due to the hydroxyl (O-H) stretching vibration [17], which is abundant due to the high percentage of starch and glycerol in the blend. An intensification in this region in the 7.5% MC films is observed, indicating the effective incorporation of the microcapsules due to the inherent chemical compatibility of the wall material with the starch. No changes in films with neat EO in this region are probably due to the lower concentration.

The range between 3000 and 2850 cm^−1^ is related to the methylene groups’ asymmetric and symmetric C-H stretching [43]. The spectra do not show significant differences for all samples. This is expected due to the absence of broken or new chemical bonds induced in the materials.

### 3.8. X-ray Diffraction—XRD

The analysis of the diffractograms in Figure 1B shows that the incorporation of microcapsules, and mainly, the neat EO, leads to the reduction in the crystallinity of the films. Corroborating this is the crystallinity index presented in Table 3, with the values ranging from 28.2 to 23.0%.

The obtained values for the crystallinity index are lower than those obtained for [17], who obtained a value of 32.3%. However, this can be due to a higher proportion of PBAT/starch (80% *w*/*w*% of PBAT) used in referred work. However, just as observed in the present work, some peaks were indistinguishable due to the overlapping of the diffraction peaks caused by the processing.

The observed peaks at 2θ = 13.2 and 20.2° are relative to the induced crystallization process of starch in type VH [44].

The EO causes a higher loss in crystallinity. A similar finding was observed in blown films of starch/PBAT with tea polyphenols incorporation, which can enhance the compounds’ phase separation or aggregation, increasing the material’s heterogeneity [17]. Related studies indicate that the free EOs added to films can act as plasticizers, as observed in cassava starch with cinnamon EO [45] and soy protein films with clove EO [46]. This is corroborated by the observed mechanical properties, which show that the low level of neat EO added promoted an improvement in tensile strength and elongation at break, followed by a decrease in these parameters with the increase in neat EO level. This is probably due to over plasticization. In all cases, the addition of neat EO promotes a reduction in the polymer’s crystallinity.

### 3.9. Scanning Electron Microscopy—SEM

**Figure 2:**
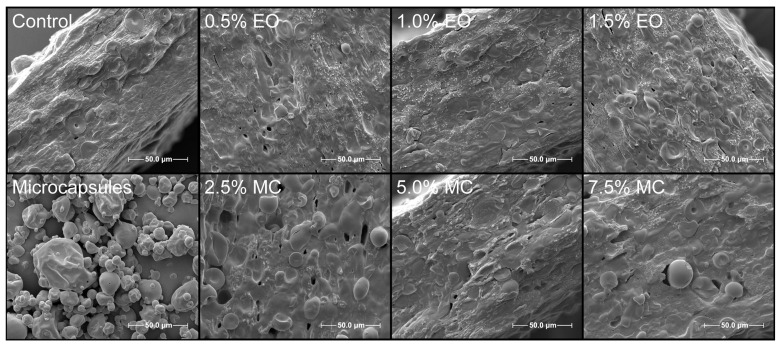
Scanning electron microscopy of the microcapsules and the active biodegradable films containing neat or microencapsulated *E. citriodora* essential oil.

The micrographs of the films and the microcapsules are presented in Figure 2. The microcapsules present a large size variability, and the surface is wrinkled; however, no cracks or fissures are observed, indicating the good protection of the essential oil that the wall material provides.

Wall materials based on higher proportions of Arabic gum (as in this work) lead to more denting due to the particles’ inflating at high temperatures and posterior shrinking. Using carbohydrates, such as maltodextrin, enhances the formation of a dry crust around the droplets, improving the drying process [47]. The selected wall material is largely used together due to the mutual advantages. Maltodextrin has a good film-forming capacity, while Arabic gum presents an adequate emulsification capacity [48], leading to better results.

Observing the film’s fracture surfaces, it is noted that all films present some porosity and some phase separation due to the inherent immiscibility of starch and PBAT, with domains with uncompleted melted starch granules. However, it is impossible to clearly identify the microcapsules in the films, as they are confounded with incompletely melted starch granule fragments.

The presence of rough surface and pores, mainly in the films with EO, can explain the reduction in the observed mechanical properties and water vapor permeability. When EO is added to pure PBAT, the surface tends to be smoother, as in hot-melt extruded PBAT films with oregano EO [49], due to the affinity of the EO with hydrophobic polymers, such as PBAT. These findings were similar to those of other researchers, such as in starch/PBAT-films containing free and microencapsulated oregano EO [19].

## 4. Conclusions

Both essential oil incorporation forms (directly or microencapsulated) could maintain antioxidant activity in the produced film. However, microencapsulation promoted higher EO protection during the film production process. The obtained films presented adequate mechanical properties with good shape, processability, and handling, making them suitable for a possible scale-up in the industry and their widespread use, demonstrating the viability of the developed film as a packaging material. According to the tests in food simulants, these films are more indicated for contact with hydrophilic non-acid or fatty foods.

## Figures and Tables

**Table 1:** Antioxidant activity by ABTS cation scavenging and total phenolic compounds in active biodegradable films containing neat or microencapsulated *E. citriodora* essential oil.

Film	ABTS (µmol of TEAC g Film^−1^)			
	Water	Ethanol 10%	Ethanol 50%	Acetic Acid 3%
MC	35.96 ± 2.45	34.32 ± 2.59	35.07 ± 0.41	23.60 ± 1.56
0.5% EO	0.92 ± 0.16 ^bC^ *	2.65 ± 0.19 ^bA^	2.74 ± 0.20 ^bA^	1.87 ± 0.04 ^cB^
1.0% EO	0.91 ± 0.27 ^bC^	3.64 ± 0.76 ^aA^	2.77 ± 0.08 ^bB^	2.13 ± 0.13 ^bB^
1.5% EO	1.23 ± 0.10 ^abD^	2.93 ± 0.39 ^abB^	3.49 ± 0.09 ^aA^	2.18 ± 0.07 ^bC^
2.5% MC	0.94 ± 0.07 ^bD^	2.41 ± 0.05 ^bB^	2.67 ± 0.03 ^bA^	2.19 ± 0.08 ^bC^
5.0% MC	1.21 ± 0.13 ^abC^	2.93 ± 0.07 ^abAB^	3.54 ± 0.59 ^aA^	2.38 ± 0.05 ^aB^
7.5% MC	1.52 ± 0.34 ^aC^	2.66 ± 0.04 ^bA^	2.95 ± 0.14 ^abA^	2.18 ± 0.06 ^bB^
**Film**	**TPCs (µg GAE g film^−1^)**			
	**Water**	**Ethanol 10%**	**Ethanol 50%**	**Acetic acid 3%**
MC	1296 ± 208	1393 ± 198	722 ± 134	894 ± 84
0.5% EO	20.36 ± 2.78 ^dBC^	21.86 ± 3.35 ^cB^	42.77 ± 2.03 ^dA^	12.72 ± 6.73 ^bC^
1.0% EO	24.75 ± 1.19 ^dB^	23.47 ± 3.49 ^cB^	54.41 ± 8.55 ^cdA^	10.44 ± 5.34 ^bC^
1.5% EO	53.41 ± 4.42 ^aB^	57.01 ± 7.28 ^aB^	142.74 ± 12.19 ^aA^	36.24 ± 3.33 ^aC^
2.5% MC	32.36 ± 2.31 ^cBC^	36.68 ± 3.29 ^bB^	53.61 ± 7.20 ^cdA^	21.46 ± 9.00 ^bC^
5.0% MC	44.69 ± 1.71 ^bB^	47.81 ± 7.18 ^abB^	74.15 ± 6.05 ^bcA^	42.16 ± 5.16 ^aB^
7.5% MC	57.52 ± 2.62 ^aB^	59.45 ± 6.06 ^aB^	93.15 ± 15.60 ^bA^	47.33 ± 2.87 ^aB^

* Values with the same lower-case letter in the same column are not different statistically (*p* > 0.05); values with the same upper-case letter in the same line are not different statistically (*p* > 0.05).

**Table 2:** Mechanical and optical properties of the active biodegradable films containing neat or microencapsulated *E. citriodora* essential oil.

Film	Tensile Strength (MPa)	Elongation at Break (%)	Young’s Modulus (MPa)	Apparent Opacity (%)	Color Difference (ΔE*)
Control	6.8 ± 0.6 ^b^ *	501 ± 53 ^ab^	42.5 ± 14.9 ^b^	64.0 ± 0.7 ^ab^	11.4 ± 0.8 ^ab^
0.5% EO	7.5 ± 0.7 ^a^	563 ± 37 ^a^	37.1 ± 7.6 ^b^	64.2 ± 0.5 ^ab^	11.4 ± 0.7 ^ab^
1.0% EO	6.1 ± 0.3 ^cd^	319 ± 65 ^d^	50.0 ± 17.0 ^ab^	63.3 ± 2.3 ^b^	10.5 ± 1.0 ^b^
1.5% EO	6.3 ± 0.4 ^bcd^	467 ± 52 ^bc^	37.7 ± 3.5 ^b^	66.6 ± 1.5 ^a^	11.4 ± 0.4 ^ab^
2.5% MC	6.8 ± 0.4 ^bc^	468 ± 69 ^bc^	50.0 ± 7.7 ^ab^	65.2 ± 1.1 ^ab^	12.7 ± 1.3 ^a^
5.0% MC	6.1 ± 0.3 ^d^	413 ± 44 ^c^	51.9 ± 10.0 ^ab^	63.4 ± 1.9 ^b^	11.8 ± 0.8 ^ab^
7.5% MC	5.7 ± 0.6 ^d^	427 ± 47 ^c^	57.7 ± 11.3 ^a^	64.3 ± 1.2 ^ab^	12.4 ± 0.3 ^a^

* Means with the same letter in the same column are not different statistically (*p* > 0.05).

**Table 3:** Weight loss in water—WLW; swelling index—SI; water vapor permeability—WVP; and crystallinity index of the active biodegradable films containing neat or microencapsulated *E. citriodora* essential oil.

Film	Swelling Index (%)	Weight Loss in Water (%)	Water Vapor Permeability (g·m^−1^·s^−1^·kPa^−1^) (×10^−10^)	Crystallinity Index (%)
Control	32.4 ± 2.0 ^a^ *	14.6 ± 0.6 ^c^	6.9 ± 0.5 ^ns^ **	28.2
0.5% EO	26.2 ± 0.9 ^bc^	14.9 ± 0.5 ^c^	6.9 ± 0.3	24.7
1.0% EO	21.9 ± 0.6 ^d^	15.6 ± 0.4 ^bc^	7.3 ± 0.4	23.0
1.5% EO	19.4 ± 1.3 ^d^	15.4 ± 0.6 ^c^	7.7 ± 0.2	24.6
2.5% MC	29.6 ± 2.0 ^a^	15.9 ± 0.4 ^bc^	6.6 ± 0.6	27.0
5.0% MC	29.3 ± 1.2 ^ab^	17.1 ± 0.9 ^ab^	6.8 ± 0.5	24.0
7.5% MC	22.7 ± 1.5 ^cd^	18.1 ± 0.6 ^a^	7.3 ± 0.3	26.9

* Values with the same letter in the same column are not different statistically (*p* > 0.05); ** ns—not significant.

## Data Availability

The original contributions presented in this study are included in the article. Further inquiries can be directed to the corresponding author.
